# Recovery of a nearly extinct Galápagos tortoise despite minimal genetic variation

**DOI:** 10.1111/eva.12014

**Published:** 2012-10-10

**Authors:** Michel C Milinkovitch, Ricardo Kanitz, Ralph Tiedemann, Washington Tapia, Fausto Llerena, Adalgisa Caccone, James P Gibbs, Jeffrey R Powell

**Affiliations:** 1 Laboratory of Artificial & Natural Evolution (LANE), Department of Genetics & Evolution, University of GenevaGeneva, Switzerland; 2 Unit of Evolutionary Biology and Systematic Zoology, Institute of Biochemistry & Biology, University of PotsdamPotsdam, Germany; 3 Galápagos National Park ServicePuerto Ayora, Galápagos Islands, Ecuador; 4 Department of Ecology & Evolutionary Biology, Yale Institute for Biospherics Studies, Yale UniversityNew Haven, CT, USA; 5 College of Environmental Science & Forestry, State University of New YorkSyracuse, NY, USA

**Keywords:** captive populations, conservation biology, conservation genetics

## Abstract

A species of Galápagos tortoise endemic to Española Island was reduced to just 12 females and three males that have been bred in captivity since 1971 and have produced over 1700 offspring now repatriated to the island. Our molecular genetic analyses of juveniles repatriated to and surviving on the island indicate that none of the tortoises sampled in 1994 had hatched on the island versus 3% in 2004 and 24% in 2007, which demonstrates substantial and increasing reproduction *in situ* once again. This recovery occurred despite the parental population having an estimated effective population size <8 due to a combination of unequal reproductive success of the breeders and nonrandom mating in captivity. These results provide guidelines for adapting breeding regimes in the parental captive population and decreasing inbreeding in the repatriated population. Using simple morphological data scored on the sampled animals, we also show that a strongly heterogeneous distribution of tortoise sizes on Española Island observed today is due to a large variance in the number of animals included in yearly repatriation events performed in the last 40 years. Our study reveals that, at least in the short run, some endangered species can recover dramatically despite a lack of genetic variation and irregular repatriation efforts.

## Introduction

In extreme cases of species endangerment, captive rearing combined with threat reduction in the wild can enable repatriation of captive-bred offspring to the original habitat and, thereby, prevent species extinction. This process has been successful in a small number of high-profile cases such as the Peregrine Falcon (*Falco peregrinus*), Arabian Oryx (*Oryx leucoryx*), Golden Tamarin (*Leontopithecus rosalia*), Guam rail (*Gallirallus owstoni*), and black-footed ferret (*Mustela nigripes*) conservation programs (Haig et al. 1990; Derrickson and Snyder 1992; Rahbek 1993; Miller et al. 1996; Ballou 1997) and had led to an increase in ex situ captive breeding programs for endangered species (Seddon et al. 2005). This said, it is also clear that rearing species in captivity reduces survival of individuals released into the wild in comparison with the success of translocating wild-caught individuals (Griffith et al. 1989; Wolf et al. 1996; Robert 2009; Johnson et al. 2010). The reduced success rate of captive breeding approaches is likely due to two types of genetic changes that occur in captivity: small population sizes and genetic adaptation to the captive environment (Allendorf and Luikart 2007; Frankham et al. 2010 and references therein). Captive breeding programs of endangered species typically start with a small number of founders. Cost and space constraints usually ensure captive populations remain small in successive generations, thus facing the negative effects associated with small population size (i.e., genetic diversity loss, inbreeding depression, accumulation of deleterious mutations; Bryant and Reed 1999; Lynch and O'Hely 2001; Charpentier et al. 2005; Xu et al. 2007; Tzika et al. 2009). Although short-term survival may not be highly correlated with genetic diversity, long-term survival most certainly is because of the potential to adapt to changing environments over long time periods is directly related to levels of genetic diversity (Madsen et al. 1999; Reed and Frankham 2003; Markert et al. 2010). For instance, the ability to survive novel disease threats that sporadically arise over long periods of time correlates with genetic diversity (Spielman et al. 2004; Sommer 2005; Smith et al. 2009). Besides small population size, another genetic change, which is specific to captive programs, is the potential for genetic adaptation to captivity to occur as a consequence of multiple generations of artificial or natural selection to captive conditions, as shown in fish, insects, and amphibians (Frankham and Loebel 1992; Lewis and Thomas 2001; Woodworth et al. 2002; Heath et al. 2003; Kraaijeveld-Smit et al. 2006). Since genetic adaptation in captivity is positively related to the number of generations in captivity, the most direct method to decrease its effect is simply to minimize the number of generations in captivity before reintroductions commence (Frankham 2008).

Here, we report on a high-profile species of giant Galápagos tortoise that has been restored to its natural habitat following a nearly 40-year-long captive breeding program. The F1 offspring of 15 breeders, the only surviving animals from the wild population, have been released into the wild rather than being retained as part of the captive breeding pool. This program provides a unique setting in which to study population genetics and survival without the added complication of genetic adaptation to captivity or matings among offspring and founders. In this case, genotyping could be performed on all parents contributing to the repatriated population, on offspring prior to repatriation, as well as on repatriated individuals surviving for varying periods of time. Moreover, because the repatriated individuals reproduced *in situ*, subsequent generations could be monitored always against the backdrop of explicit knowledge of the genetic makeup of the founding population.

During his visit to the Galápagos in 1835, Darwin was informed that the giant Galápagos tortoises occupying various islands were morphologically distinct (Darwin 1839); observing this diversity was important to Darwin's conception of evolution by natural selection. While the taxonomy/systematics of these tortoises has a long and convoluted history, 11 of the 15 recognized species still exist, although eight of them are threatened with extinction (Van Denburgh 1914; Pritchard 1996; Le et al. 2006). One species of Galápagos tortoises became extinct recently: ‘Lonesome George’, the last representative of the Pinta Island tortoise (Chelonoidis abingdoni) died on June 24, 2012. The island of Española previously hosted a morphologically distinct and endemic species of tortoise (*Chelonoidis hoodensis*). Originally numbering in the thousands on the island of just 60 km^2^, by the 1960s, tortoises were rarely seen and those found were all mature adults that evidently had not bred for an extended period (or had produced offspring that did not survive). Initial decimation of this species was likely due to exploitation by whalers; destruction of habitats by human-introduced goats was the immediate threat. Efforts made to locate all remaining tortoises on the island yielded twelve females and two males. In 1977, a male from the San Diego Zoo was added to the breeding program initiated at the Galápagos National Park Service (GNPS) headquarters on the island of Santa Cruz (Bacon 1978; Fritts 1978; Cayot and Morillo 1997). All indications are that these 15 breeders are indeed the only survivors of this species, that is, there is no evidence that any native tortoises were missed. Eradication of goats on Española Island was completed in 1978, leading to rapid recuperation of the vegetation. Beginning in 1975 with tortoises born in captivity in 1971, offspring descended from the 15 survivors have been repatriated periodically to Española Island after reaching about 20 cm in size or typically 5 years of age. To date, 1767 offspring have been released. At present, tortoises occupy about one-third of the suitable habitats available to them on the island.

In 1994, we collected blood samples from the 15 parents in captivity and 134 surviving F1 tortoises on Española Island and demonstrated that the population is exceptionally low in genetic variation. More specifically, it exhibits a single maternal lineage (as assessed by mitochondrial DNA; Caccone et al. 2002) as well as a very low number of alleles and mean expected heterozygosity at microsatellite loci (Ciofi et al. 2002; Milinkovitch et al. 2004). Here, using blood samples from an additional 311 and 214 individuals collected on Española Island in 2003 and 2007, respectively, we describe the outcome of the breeding program by determining parentage of the repatriated animals and by searching for genetic signatures of *in situ* reproduction. As the samples collected on Española Island do not allow us to distinguish nonrandom breeding from differential survivorship after release, we additionally collected, in 2003 and 2007, blood samples from 154 and 227 juvenile tortoises, respectively, in the breeding center before their release to Española Island.

## Methods

Blood samples were collected from the brachial vein, preserved in a lysis buffer (0.1 m Tris–HCl, 0.1 m EDTA, 0.2 m NaCl, and 1% SDS) at room temperature and a pH of 8.0. About 200 μL of each blood sample was digested at 37°C overnight in a buffer (100 mm of Tris–HCl, 100 mm of NaCl, 5 mm of EDTA, 0.5% SDS) containing 200 μg of proteinase K. Genomic DNA was isolated following standard phenol–chloroform extraction procedures, and DNA was resuspended in Tris–EDTA (TE) buffer (10 mm of Tris–HCl and 1 mm of EDTA at a pH of 7.2) and stored at −20°C. Multiplex genotyping was performed at 15 loci as in (Milinkovitch et al. 2004) using three fluorescent dyes and ‘HotStarTaq Plus’ polymerase (Qiagen, Venlo, the Netherlands) with the following thermal profile: 95°C 5-min activation step, followed by 28 cycles of denaturation at 95°C for 30 s, annealing at 60 or 62°C for 90 s, and extension at 72°C for 30 s. A common DNA polymerase characteristic is to sometimes add an Adenine at the ends of PCR products generating (for each allele) a mix of products differing in lengths by one nucleotide (‘−A’ and ‘+A’ products). Different ratios of +A/−A are obtained depending on specific templates, PCR conditions and primers, complicating interpretation of fragment analysis data. To avoid such problems, we added a GTTTCTT tail in 5′ of the unlabeled primer and used a final extension step of 60 min at 72°C at the end of the genotyping PCR. This procedure greatly favors +A products and thereby improves binning of alleles. PCR products were sized using an ABI3100 capillary sequencer with GeneScan 350 ROX Size Standard (Applied Biosystems, Foster City, CA, USA). Raw data were analyzed with Genemapper v3.7 (Applied Biosystems) with further correction of allele binning with TANDEM v1.09 (Matschiner and Salzburger 2009). The targeted loci had been previously selected as the most informative for maternity and paternity analyses after screening the breeders for variation at 69 microsatellite loci and excluding loci generating null alleles or linkage disequilibrium (Ciofi et al. 2002; Milinkovitch et al. 2004). Maximum likelihood parentage analyses were performed with PASOS v1.0 (Duchesne et al. 2005), allowing for the presence of nonsampled parents (for the identification of F2 individuals and potential contaminations from non-Española parents; Milinkovitch et al. 2007). Any individual sampled on the island was identified as F2 when its mother or father or both could not be identified among the 15 breeders but all alleles were found in the parental population. We estimated the sex-ratio-corrected effective population size according to Wright (Wright 1931) after additional correction for differential reproductive success (identified through parentage analyses) using equations from the study by Lande and Barrowclough (1987). The microsatellite data is provided in Table S1 with the 15 locus genotypes of all individuals.

To estimate the age distribution function of repatriated tortoises, the curved length of the carapace (i.e., the distance from the nuchal to the supracaudal scutes along the carapace) was measured for each animal sampled on Española Island in 2007. The frequency distribution of carapace sizes was plotted using bin sizes of 4 cm. We also simulated expected frequency distribution of carapace sizes using the available data on the number of juveniles included in each of the 28 repatriation events documented between 1970 and 1991. The carapace curve length at release was assumed to be about 20 cm.

## Results and discussion

**Figure 1:**
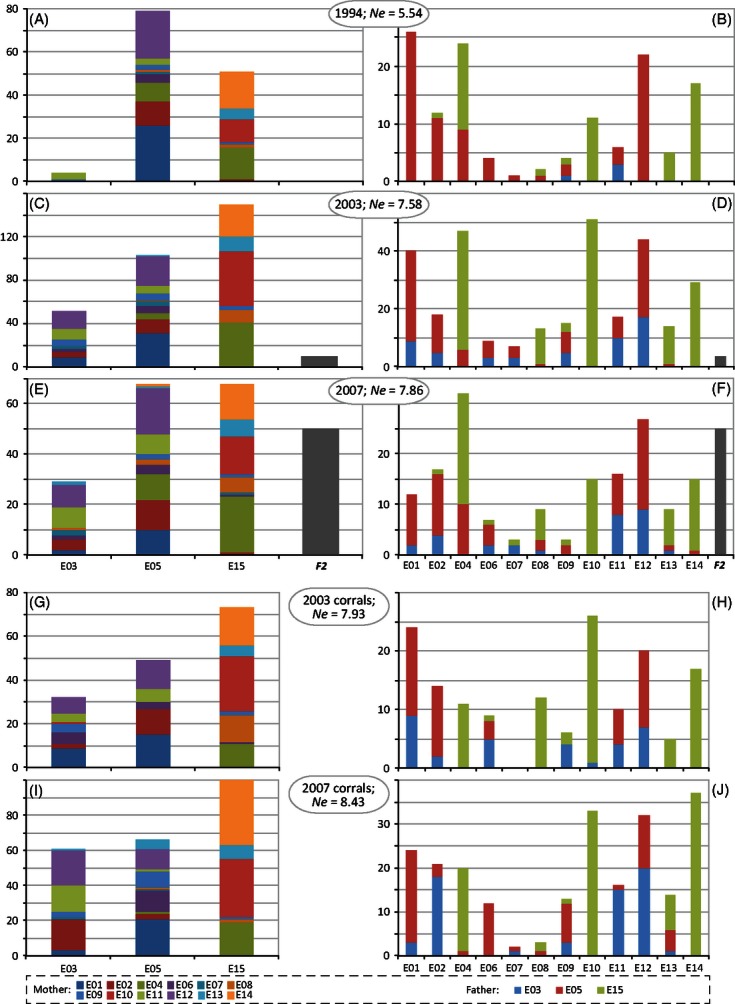
Distribution of offspring assigned to three breeding males and 12 breeding females for samples collected on Española in 1994 (A, B), 2003 (C, D), and 2007 (E, F) as well as before release in 2003 (G, H) and 2007 (I, J). F2; individuals conceived and hatched on the island.

Contribution of the breeders to offspring surviving on Española island has been, and continues to be, uneven among individuals (Fig. 1). Taking this into consideration, we estimated the effective population size (*N*_e_) of the repatriated population to be 5.5, 7.6, and 7.9 in the 1994, 2003, and 2007 samples, respectively (Fig. 1a–f). The parental contributions to prerelease individuals are similar to those surviving in the field (Fig. 1g–j), indicating that unevenness of genetic contribution to survivors in the repatriated population is mostly due to differential reproductive success of the breeders and/or nonrandom mating in captivity.

Has this captive breeding-repatriation program been a success? In other words, is there evidence of substantial breeding *in situ* by repatriates? By 1990, tortoise nests were observed on Española Island, and at least one offspring was documented by us in 1994. We show here (Fig. 1a–f) that the number of F2 individuals sampled on Española Island increased from 0 in 1994 to 9 (2.6%) in 2003 and to 50 (24%) in 2007. Fully one-quarter of the population now are offspring of repatriates, and that fraction is growing rapidly. Hence, the repatriation program has clearly been a success in the context of establishing a selfreproducing population among the repatriated individuals and restoring the species ecological role as the largest herbivore on the island.

**Figure 2:**
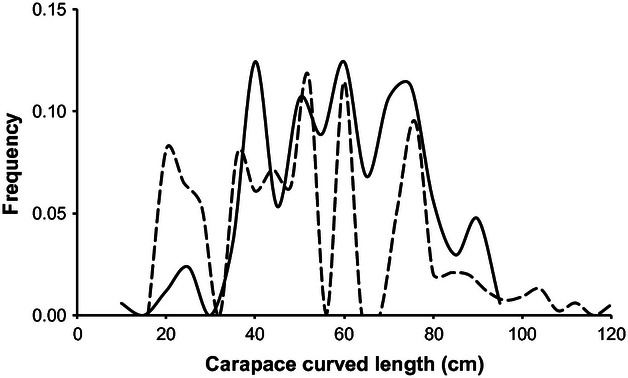
The heterogeneity in the rate of released individuals in the last 40 years explains most of the distribution of tortoise sizes on Española observed today. Expected (dotted line) and observed (plain line) frequencies of carapace size classes. Observed and expected distributions are highly significantly correlated: parametric Product-Moment-Correlation Coefficient (assumes Normal-distributed carapace size) = 0.580, df = 33, *P* < 0.001; nonparametric Spearman-Rank-Correlation Coefficient (does not assume any particular distribution of carapace size) = 0.505, df = 33, *P* = 0.003.

We also performed basic morphological measurements on all tortoises sampled on Española Island to roughly estimate potential age-related mortality. We observed the presence of about six cohorts of sizes (Fig. 2, plain line). Although young tortoises had been repatriated very regularly (one to two campaigns of repatriations have typically been performed each year), analysis of objective repatriation data allowed us to identify a large variance in the number of animals released each year. We, therefore, performed growth-mortality simulations using the available data on release dates and number of released individuals. Assuming, for all repatriated animals, a release size of 20 cm, a 5% annual mortality rate and an annual growth rate of 4 cm, we obtained an expected distribution of carapace sizes in the population of 2007 (dotted line in Fig. 2). The simulated and observed size distributions are highly significantly correlated (see Fig. 2 for details). This analysis indicates that most of the heterogeneity in the distribution of tortoise sizes on Española Island observed today is simply explained by the corresponding heterogeneity in release efforts in the last 40 years, without the need for involving differential mortality rates among age classes or environmental parameters affecting growth rates among years.

Our study reveals that some endangered taxa can recover dramatically despite a lack of genetic variation and irregular repatriation efforts. One possible reason for this success is that the breeders are the survivors from the original natural population. No long-term selection over multiple generations for adaptation to captive breeding has been imposed on the breeders, a factor that has led in other breeding programs to reduced success of released offspring back into nature (Williams and Hoffman 2009). However, we emphasize that given the long generation time of these tortoises (∼25 years to first reproduction), the Española Island captive breeding-repatriation program is in its very early stages despite being four decades in the making. On the other hand, both theoretical considerations and empirical studies suggest that the level of genetic diversity of endangered species is positively correlated with long-term persistence (including surviving introduced diseases) and long-term adaptation (Madsen et al. 1999; Reed and Frankham 2003; Markert et al. 2010). Another attribute of the long-term viability of populations is the ability to recover from population ‘bottlenecks’, which is directly related to the intrinsic rate of population growth and duration of bottleneck. Recent microsatellite analyses suggest that Española tortoises have been subjected to population bottlenecks at different points in time after and prior to human arrival in the archipelago (Garrick et al. 2012). Given the estimations of an Española population in the thousands only 100–300 years ago (4–12 generations) (Townsend 1925; Pritchard 1996), this implies that historically Española Island tortoises may have been subjected to population declines not directly associated with human activities. These abrupt declines could be related to the fact that Española is the lowest island with a native tortoise population, making it more subject to environmental fluctuations, mainly extreme weather events that affect carrying capacity, fires, and tsunamis. Even slight reduction in birth rate due to lack of genetic diversity may impede population or species recovery from a bottleneck leading to extinction (Nei et al. 1975). In contrast, these bottlenecks may have led to purging of deleterious recessive alleles improving the long-term survival prospects of the repatriated population (Facon et al. 2011; Garnett and Zander 2011).

Thus, the long-term survival prospects of the Española tortoise remain uncertain and could be improved by increasing the capture of remaining genetic diversity of the breeders within the repatriated population. Even though our molecular genetic studies did not begin until nearly 25 years into this captive rearing program, all breeders remain alive and still actively contributing to the offspring pool, so there is still opportunity to adjust captive breeding regimes. Molecular-marker-assisted selection of most-divergent mating partners and release of under-represented genotypic combinations is one option (Jones et al. 2002; Tzika et al. 2009; Henkel et al. 2012). However, the relative frequency distribution of pairwise genetic identities among the 15 Española Island breeders is unimodal (Fig. 2 in Milinkovitch et al. 2004). The discriminating power of this approach (to conclude for the absence of particularly closely related individuals) is low because of the high background genetic identity generated by the exceptionally low genetic variation of the Española Island population. Clearly, a better assessment of the genetic relatedness among breeders would require many more loci than those 15 used here. In addition, computer simulations (Ivy et al. 2009) suggested that the incorporation of molecular estimates of relatedness into management of populations might provide little benefit when the number of related founders is small. It might, therefore, be more efficient to assume breeders are unrelated and attempt to reduce inbreeding in the repatriated population by better equalizing reproductive success among breeders. This is, however, not necessarily simple to achieve in practice because of logistic and economic considerations that weigh heavily on the management of this breeding program. Moreover, tortoises are not indiscriminate breeders and so will not necessarily breed if simply placed together. Although the exact history of tortoise pairing in captivity is incomplete, it is certain that males and females have had unequal access to mating partners during the breeding program. We are currently attempting to establish new breeding groups where each male would be associated with the set of females which he had little or no access to in the past. The reproductive output of each female would then be assessed in the future to determine periodical swaps of individuals among breeding groups to maximize retention of what little genetic variation remains.

The captive breeding/repatriation program for the species of giant Galápagos tortoise endemic to Española Island has been ongoing for four decades. It could be argued that the program has experienced limitations: (i) the strong heterogeneity in breeding contribution of the breeders has reduced effective population size, (ii) the repatriation output has been irregular, and (iii) a Pinzón juvenile male has likely been accidentally incorporated into an early Española release (early 1970s) and generated at least one transisland (Pinzón/Española) hybrid on Española island two decades later (Milinkovitch et al. 2007). Much more compelling have been the successes of the *Chelonoidis hoodensis* captive breeding/repatriation program: (i) survivors on Española island have been located and repatriated to a breeding facility, (ii) a worldwide search identified the presence in a US zoo of a male originating from Española island, and this individual has been added to the breeding program in the Galápagos, (iii) the habitat restoration process has progressed on Española Island after successful eradication of feral goats (the primary cause for habitat destruction), (iv) nearly 2000 captive-born offspring have been released on Española Island, where they currently occupy one-third of the suitable habitats, (v) *in situ* reproduction is now substantial and increasing, and (vi) discussions have been initiated for the establishment of new breeding groups for increased effective population size of the repatriated population. Hence, this captive breeding/repatriation program has not only saved a high profile and endemic species of giant Galápagos tortoises from extinction, but enabled it to experience a seemingly spectacular and ongoing recovery *in situ*. Moreover, guidance from molecular genetic analyses has been critical in achieving these successes.

## Data archiving statement

Raw data for this study are available as online supplementary materials (Table S1).

## Supporting Information

**Table S1.** Microsatellite data (at 15 loci) for all individuals used in this study.
